# Feasibility of Motivationally Enhanced Compensatory Cognitive Training for Older Veterans With MCI

**DOI:** 10.1093/geroni/igaf122.3934

**Published:** 2025-12-31

**Authors:** Kate Shirley, Laura Campbell, Jacqueline Maye, Emily Sano, Delaney Pickell, Amy Jak, Maya O’Neil, Elizabeth Twamley

**Affiliations:** Brown University, Providence, Rhode Island, United States; University of California, San Diego, San Diego, California, United States; VA San Diego Health Care System, San Diego, California, United States; VA Portland Health Care System, Portland, Oregon, United States; VA San Diego Health Care System, San Diego, California, United States; VA San Diego Health Care System, San Diego, California, United States; VA Portland Health Care System, Portland, Oregon, United States; VA San Diego Health Care System, San Diego, California, United States

## Abstract

Mild Cognitive Impairment (MCI) increases risk for functional decline and dementia, yet effective interventions for older adults remain limited. Motivationally Enhanced Compensatory Cognitive Training (ME-CCT) is a manualized, 8-week cognitive rehabilitation group intervention delivered in-person and via telehealth that integrates compensatory cognitive strategies, motivational interviewing, and lifestyle education. In this multisite randomized controlled trial, 156 older Veterans (Mean age = 71.5, SD = 7.7) with MCI were randomized to ME-CCT (n = 84) or a Goal-Focused Supportive Contact control condition (SC; n = 72). Among participants who attended at least one session, mean attendance was high in both groups (ME-CCT: 7.7 ± 1.3 of 8 sessions; SC: 7.5 ± 1.7), with few never attending (ME-CCT: 5; SC: 6). Dropout among those who started was low and comparable across groups (ME-CCT: 5.1%; SC: 7.6%). Session attendance for ME-CCT was consistent across delivery formats, with in-person participants (n = 63) attending 7.7 ± 1.2 sessions on average and telehealth participants (n = 21) attending 7.5 ± 1.7 sessions. Dropout rates were also comparable across delivery formats (In-person: 8.3%; Virtual: 10.5%). ME-CCT was well received: 96.9% rated quality as good to excellent, 92.2% reported improved problem management, and 95.2% were satisfied with services. Findings indicate ME-CCT is feasible and acceptable for older Veterans with MCI across delivery formats, with high attendance, low dropout, and strong satisfaction. Future research should prioritize optimizing telehealth delivery and evaluating whether sustained engagement yields cognitive and functional benefits.

